# Postmastectomy Radiation Therapy: Are We Ready to Individualize Radiation?

**DOI:** 10.1155/2018/1402824

**Published:** 2018-03-01

**Authors:** Ashlyn S. Everett, Drexell Hunter Boggs, Jennifer F. De Los Santos

**Affiliations:** ^1^Department of Radiation Oncology, University of Alabama at Birmingham, Birmingham, AL, USA; ^2^Hazelrig Salter Radiation Oncology Center, 1700 6th Ave South, Birmingham, AL 35249, USA; ^3^The Kirklin Clinic at Acton Road, 2145 Bonner Way, Birmingham, AL 35243, USA

## Abstract

Contemporary recommendations for postmastectomy radiation have undergone a shift in thinking away from simple stage based recommendations (one size fits all) to a system that considers both tumor biology and host factors. While surgical staging has traditionally dictated indications for postmastectomy radiation therapy (PMRT), our current understanding of tumor biology, host, immunoprofiles, and tumor microenvironment may direct a more personalized approach to radiation. Understanding the interaction of these variables may permit individualization of adjuvant therapy aimed at appropriate escalation and deescalation, including recommendations for PMRT. This article summarizes the current data regarding tumor and host molecular biomarkers* in vitro* and* in vivo* that support the individualization of PMRT and discusses open questions that may alter the future of breast cancer treatment.

## 1. Introduction

Breast cancer is the most commonly diagnosed malignancy and the second most frequent cause of cancer death in women [1]. Radiation therapy remains an important treatment modality for patients with locally advanced breast cancer, with randomized trials investigating postmastectomy radiation therapy (PMRT) continuing to demonstrate decreased locoregional recurrence (LRR) and improved overall survival (OS) in appropriately selected patients [2–4]. Historically, surgical staging provided risk-stratification that served as the basis for recommending PMRT; however, modern understanding of genetics, immunology, and molecular biology is shedding light on tumor and host biomarkers that may alter patients' rates of response, progression, or survival [5].

Precision radiation therapy currently refers to the use of advanced technology to improve target delineation and treatment delivery. However, to usher radiation oncology into the era of personalized medicine, we must consider individual patient and tumor parameters that influence treatment response and complications to provide appropriate treatment recommendations. Applying our understanding of molecular markers representing tumor and host biology may permit individualization of radiation therapy aimed at appropriate escalation and deescalation of PMRT based on biologic parameters. This article summarizes the current data regarding molecular markers, which support the individualization of radiation using tumor molecular profiles, stromal microenvironment data, and patient genetic and immune parameters.

## 2. Tumor Molecular Profile

### 2.1. Tumor Biology

In the past decade, great strides have been made in understanding tumor biology and using this knowledge to design tumor-directed targeted therapies. For example, the addition of targeted monoclonal antibody therapies against the HER2 protein (i.e., trastuzumab, pertuzumab) to chemotherapy regimens has significantly improved overall survival in patients with HER2 amplified breast cancers [6]. However, heterogeneity within individual tumors significantly limits targeted therapies potential to cure cancer, as we have recognized tumors developing evasion mechanisms and resistance to targeted treatment strategies.

In breast cancer, tumor heterogeneity, as represented by receptor status, has been recognized for decades. More recently, genomic assays of 12–70 or more genes, including Oncotype Dx (Genomic Health, Inc., USA, Redwood City, CA) and MammaPrint (Agendia, Inc., USA, Irvine, CA), have further elucidated the heterogeneity of breast tumors. Medical oncologists apply this data in making treatment recommendations regarding adjuvant chemotherapy; however, little data relates this genomic data to radiation oncology recommendations. While genomic models provide primarily information regarding distant failure rates, as opposed to local failure rates, several investigators have explored the applicability of existing recurrence scores to the issue of locoregional recurrence [7].

Some studies propose models accounting for intrinsic radiosensitivity of tumors to help guide radiation recommendations and dose schedules. Eschrich and colleagues validated a radiosensitivity molecular signature in a cohort of breast patients, which demonstrated that patients predicted to be radiosensitive versus radioresistant had improved 5-year relapse-free survival and 5-year distant metastasis-free survival when treated with radiation [8]. A similar model was used to predict local recurrence in breast cancer and identified that radiosensitive triple-negative phenotype breast cancers had similar local recurrence to those with luminal A or B subtypes [9]. While there is no defined role for these clinical models at this time, they potentially can provide clinicians with valuable information regarding intrinsic tumor radiosensitivity and biology that may shape clinical recommendations in the future.

### 2.2. Genomic Factors

While genetic mutations in tumors are widely recognized, epigenetic changes are now appreciated to influence response to radiation treatment across many cancer histologies, including breast cancer [10]. DNA methylation is a process involved in regulating gene transcription, and, therefore, gene expression and cellular function [11]. Data suggests a correlation between radiosensitivity and DNA methylation* in vitro* [12], and* in vivo* studies demonstrate DNA methylation levels of specific genes* before and after* exposure to radiation are significantly correlated with response to radiotherapy and with total dose [13]. In clinical context, using methylation of particular genes to predict treatment response may help physicians tailor radiation therapy using individual patients' genomic DNA methylation pattern.

## 3. Stromal Microenvironment

The tumor microenvironment is a known factor influencing radiation treatment and response but has proven difficult to study, given our limitations in reproducing its conditions* in vitro*. Several recent studies have demonstrated molecular influences of the stroma on irradiated tissues. When comparing tumor cells irradiated* in vitro* versus* in vivo*, gene expression profiles after radiation differed significantly, with authors concluding that the tumor microenvironment (accounting for hypoxia, nutrient availability, blood flow, etc.) plays a major role in gene expression after radiation [14]. Similarly, studies investigating DNA methylation of breast cancer show that methylation changes* in vitro* are significantly lower than the methylation seen in patient samples, suggesting a significant impact of the tumor microenvironment and host immune system [13].

There is also striking difference between gene expression after single fraction dose radiation versus multifraction dose radiation, with multifraction dose radiation demonstrating increased expression of IFN-related genes. IFN-related genes are known to contribute to inflammation and be associated with radiation resistance that is often associated with multifraction radiation schedules [14]. This data suggests that inflammation in the microenvironment around the time of radiation may influence the response to radiation therapy and could be a potential target to increase the therapeutic ratio of radiation in future studies. Other studies investigating the stromal microenvironment demonstrate that genetic changes, notably in the* c-Kit* and ER*α* proteins, are present and continually changing years after radiation therapy, likely contributing to fibrosis, telangiectasias, and other normal tissue complications [15]. While significant questions remain, there is now concrete data demonstrating that tumor microenvironment plays a substantial role in influencing the effect of radiotherapy and may play a part in individualizing radiation therapy in the future.

## 4. Host Parameters

### 4.1. Genetic Factors

Host genetic factors are known to play a role in development of cancer. Genetic mutations are of two varieties: germline genetic mutations, arising from the gamete and therefore affecting every cell in the offspring patient, and somatic mutations, occurring in a population of cells after conception and often found in tumor cells [16, 17]. Patients harboring germline mutations, such as BRCA1 or BRCA2 and TP53 (Li-Fraumeni syndrome) or ATM, are often at higher risk of developing cancers, since every cell in the body has an existing mutation.

In contrast, somatic mutations are not known to confer higher risks of developing future cancers, since the known defect is limited to a certain population of cells. Using known genetic risk information to assist in making treatment recommendations in breast cancer is crucial for optimizing patient outcomes. In general, adjuvant radiation therapy risks and benefits should be discussed in a multidisciplinary setting, with an understanding of treatment-related complications. Here we will briefly discuss two genetic syndromes that are relevant with regard to adjuvant radiation therapy.

#### 4.1.1. Li-Fraumeni Syndrome

Patients with Li-Fraumeni syndrome have germline TP53 mutations, and significant risk of early onset breast cancer, the most common malignancy diagnosed in adults with this mutation [18]. With modern understanding of this genetic mutation, there is an appreciation for increased risk of second cancers, including sarcoma, leukemia, and second breast primaries (ipsilateral and contralateral) [19]. Radiation therapy is generally avoided in patients with Li-Fraumeni syndrome but should be discussed with a multidisciplinary team, considering the risks and benefits of disease recurrence with complications including second malignancies.

#### 4.1.2. ATM

Mutation in the ataxia telangiectasia mutated (ATM) gene is another one that confers increased risk of breast cancer. Homozygous ATM mutations also results in potentially increased toxicity with radiation therapy, due to defective DNA repair and genomic instability in normal tissues [20]. However, patients with heterozygous germline ATM mutations do not appear to have increased toxicity with radiation therapy [21]. Radiation therapy is generally contraindicated in patients with homozygous germline ATM gene mutations but appears to be safe in appropriately selected patients with heterozygous mutation [22].

### 4.2. Host Immune Profile

For over 40 years, the immune system has been implicated as crucial for optimal response to radiation therapy [23]. Modern studies show that DNA methylation of immune pathway genes is significantly altered after radiation therapy, consistent with an influence on response to radiation [13]. In addition, upregulated inflammatory signaling pathways are implicated in improving response to radiation and may be promising targets for enhancing radiosensitivity of tumors [24]. Current immunotherapies in use for other cancer types include ipilimumab, pembrolizumab, and nivolumab. Trials incorporating immunotherapies neoadjuvantly are currently underway in high risk breast cancer. One such study, the I-SPY 2 trial (http://www.ispy2.org), investigates the genomic profile of breast tumors using the MammaPrint Assay (Agendia, Inc. USA, Irvine, CA). Patients with high risk results on the MammaPrint are then randomized to various arms of neoadjuvant chemotherapy, with one arm including pembrolizumab (anti-PD-1 antibody). Preliminary results in the pembrolizumab arm demonstrate increased pCR rates (TNBC 20 versus 60%, HER2+ 13 versus 35%) compared to the standard chemotherapy arm [25]. These are promising results and will hopefully translate into improved outcomes with further follow-up and may open the door for incorporating immunotherapies into the standard chemotherapy regimen for locally advanced breast cancers.

## 5. Current Strategies for Individualizing Radiation

Researchers from Moffitt Cancer Center recently proposed one method of adjusting radiation dose using biological differences in tumors. The genome-based model for adjusting radiotherapy dose (GARD) incorporates gene-expression-based radiation sensitivity, standard of care radiation dose, and fractionation for a particular tumor and the linear quadratic model to produce a value which predicts for the therapeutic effect of radiotherapy. In short, a higher GARD value predicts a higher therapeutic effect of radiation, which could be extrapolated to clinical benefit. Retrospective cohort studies using the GARD model have stratified patients into low, middle, and high groups, demonstrating that GARD may be an independent predictor of clinical outcome in breast cancer patients. In multivariate analysis, distant metastasis-free survival and relapse-free survival in multiple cohorts were statistically associated with GARD values. Authors discussed that GARD may be used as a method to customize radiation dose to match the radiosensitivity of individual patient's tumors, paving the way for future clinical trial design. However, limitations of GARD include the fact that it does not account for host or microenvironmental factors, or normal tissue toxicity factors, which, if included, would further allow clinicians to optimize radiation dose [26].

## 6. Conclusion

In the past decade, we have seen a paradigm shift in the treatment of breast cancer, with a more individualized approach to understanding tumor biology and recommending adjuvant chemotherapy. Moving into the next decade of cancer care, it is imperative that personalized medicine moves beyond medical oncology and includes radiation therapy. This will present many challenges, requiring radical changes to the traditional model of delivering radiation—which has been to deliver the high dose radiation therapy within normal tissue tolerances.

Clinicians must synthesize our current understanding of tumor biology, stromal microenvironment, host genetic and immune factors, risk of recurrence and complications, and improved therapeutics to make up-to-date and appropriate recommendations for our patients. Ongoing research may enhance our understanding of factors that may be incorporated into future clinical trials, testing various dose levels based on the genetic, genomic, immune, stromal, and tumor molecular profiles discussed in the body of this paper. Future studies may include similar models to GARD, where combinations of tumor factors, tumor microenvironment, host immunity, and normal tissue complications and host genetics and immune profiles are implicated in designating radiation dose levels. Ultimately, precision radiation therapy must begin to incorporate personalized treatment recommendations, eliminating use of radiation in patients who do not benefit from it and preferentially treating those with highest benefit, to optimize breast cancer patient outcomes and improve our quality of care.

## Additional Points

*Key Points. *(i) Current understanding of genetic, genomic, immune, stromal, and tumor molecular profiles may be useful in providing a strategy for bringing personalized medicine into the field of radiation oncology. (ii) Precision radiation therapy should eliminate the use of radiation in patients who do not benefit from it and preferentially treat those with highest benefit, to optimize breast cancer patient outcomes.
